# Analysis of Provincial Mortalities Among Bus/Minibus Users Over Twelve Years, East Azerbaijan, Iran

**DOI:** 10.25122/jml-2018-0051

**Published:** 2018

**Authors:** Sadeghi-Bazargani Homayoun, Samadirad Bahram, Golestani Mina, Shahedifar Nasrin, Jamali Milad

**Affiliations:** 1. Road Traffic Injury Research Center, Statistics and Epidemiology Department, Tabriz University of Medical Sciences, Tabriz, Iran; 2. Legal Medicine Research Center, Legal Medicine Organization, Tehran, Iran; 3. Road Traffic Injury Research Center, Tabriz University of Medical Sciences, Tabriz, Iran; 4. Statistics and Epidemiology Department, Tabriz University of Medical Sciences, Tabriz, Iran

**Keywords:** epidemiology, injuries, mortality, bus, minibus, road traffic accident

## Abstract

**Objective:** The aim of this study was to investigate the epidemiological features of bus/minibus users’ road traffic injury mortalities during 2006-2017, in the East Azerbaijan province of Iran.

**Methods:** All 245 bus/minibus users’ mortalities, registered in the forensic medicine database, were analyzed by STATA 13 statistical software package.

**Results:** The majority of victims (mean age: 41.5±18.6 years) were men (70%), adults (79.18%), illiterate (22.4%) and self-employed (25.3%). Passersby and police played an almost null role in transporting victims since 2014. A decreasing trend of bus/minibus users’ fatalities was observed over the study time. Head-neck-face trauma was more common among those who died prior to hospitalization. Rollover was significantly prevalent among bus users and falling among minibus users. Lorries, vans, and trailers as crash counterpart vehicles caused 59% of deceases, excluding the cases when no other vehicle was engaged. Victims were more likely to die at the hospital when crashes happened in the city’s inner roads (OR: 4.17; 95%CI:1.7-9.9). The elderly were 2.78 times more likely to die at the hospital when compared to the other age groups (95%CI: 1.23-6.26).

**Conclusions:** To identify a target group for interventions on traffic-related knowledge, attitude and behaviors, male adults, illiterate and self-employed bus/minibus users could be of priority. Type vehicles involved in the crash should be considered as an important factor affect on crash fatalities. Further investigations are needed in this regard in the future.

## Introduction

As one of the most significant challenges of healthcare, Road Traffic Injuries (RTAs) globally cause 1.3 million deaths annually and contribute to mental, physical and financial problems [1, 2]. Regarding the number of mortalities caused by RTAs, Iran is the top 5th country in the world and is the first in the Eastern Mediterranean region. The mortality risk for passengers of cars, minibuses and buses are at least twice as high as truckers. The mortality risk in the bus and minibus categories is higher due to the greater number of passengers and drivers and passengers not using seatbelts when compared to other vehicles. Also, the absence of standard norms for vehicles is another issue that requires further investigations in terms of vulnerability problems in low and middle-income countries (LMICs) [3].

In Iran, based on the national forensic medicine database [4], during a five-year period, 13 collisions related to buses caused 919 deaths. This significant statistic certainly indicates the necessity of in-depth investigation exclusively on collisions related to vehicles of public transportation like buses and minibuses. More precisely, despite of much research done on the road traffic-related concerns, detailed studies on public transport traffic-related injuries are scarce, particularly in the North-West of Iran [5]. Thus, collective evidence demonstrates a primary necessity of figuring out the multifactorial aspects of mortalities among bus and minibus users. The current paper provides the latest epidemiological data on the bus/minibus users’ (BuMUs) mortalities, over twelve-years between 21 March 2006 and 20 March 2017 in the East Azerbaijan province of Iran.

## Methods

This cross-sectional study was led in the East Azerbaijan province of Iran. East Azerbaijan province is located in Northwest Iran. According to the recent census in 2011, 5% of the country population equal to almost 3,725,000 people live in East Azerbaijan [6]. All forensic medicine centers are authorized to inspect all road traffic fatalities for 30 days after a crash, after which all the data are submitted to the central forensic medicine organization in Tabriz, the provincial capital city of East Azerbaijan.

### Selection and Description of Participants

All over the province, all 245 bus/minibus users’ fatalities (2.22%) owed to RTAs which were registered between 21 March 2006 and 20 March 2017 (Persian calendar: Farvardin 1, 1385- Esfand 29, 1396) in the East Azerbaijan Forensic Medicine Database (EAFMD) were included in the study. Here, by the “bus/minibus user” we mean bus/minibus driver or passenger.

In the current paper, motor vehicles are defined based on Iranian national traffic codes. Accordingly, vehicles are classified mostly based on the capacity of total interior passengers and cargo volumes which is as follows:

**Car:** a motor vehicle for the conveyance of maximum 6 persons designed to transport people only, including brands such as Peugeot 405, Peugeot 206, Pride, Peugeot Pars, Samand, Peykan, and L90.

**Autobus:** a motor vehicle for the conveyance of at least 27 people including its driver, driver’s assistant and passengers. The brand names considered by the forensic medicine organization data collection guidelines include Benz, Shahab, Volvo, and Scania.

**Minibus:** a passenger vehicle with the capacity of 16 to 26 seats including its driver. No brands are listed for this category.

The schooling level of preschool age victims was defined as “illiterate” and their job as “others”. More precise information on crash- and victim-related data, as well as the research methodology in detail, are published and accessible at the protocol of method and related papers [4, 7]. The jobs were categorized into 14 groups including school students, university students, housekeepers, employees, workers, self-employed, soldiers, retired and jobless people, drivers, farmers, military, other jobs, and unknown. The classification is presented according to the questionnaire of Forensic Medicine Organizations.

### Statistics

Using STATA 13 statistical software package (STATA Corp, Texas), descriptive statistics such as frequency, mean, standard deviation (SD), odds ratios (OR) and 95% Confidence Intervals (95% CI) of effect size were calculated. Inferential statistics like Chi-squared test and multivariate logistic regression were also applied to assess potential associations between categorically scaled variables and hospital fatality (P-value < 0.05). A P-value below 0.1 was considered in selecting the variables to be introduced into the multivariate regression model.

The study protocol was approved by the joint research committee of Forensic Medicine and Research Center for Road Traffic Injury, as well as the Regional Ethics Committee of Tabriz University of Medical Sciences (TUOMS).

## Results

Of the entire 11016 Road Traffic Injury (RTI) deaths registered in the East Azerbaijan Forensic Medicine database from 21 March 2006 to 20 March 2017, 245 fatalities (2.22%) were bus and minibus users who were included in this study.

**Figure 1:**
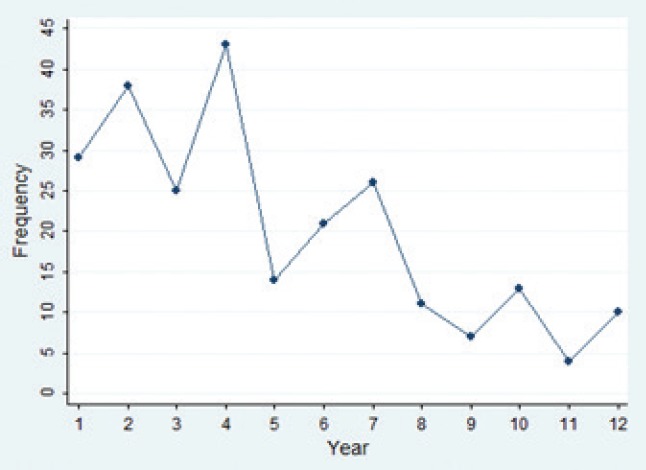
The trend of road traffic fatalities among bus/minibus users, East Azerbaijan, Iran (March 2006-March 2017) *Here, 1 represents the year of 2006, 2 represents the year of 2007 and so on.

A decreasing trend of fatal traffic crashes was observed among bus and minibus users over the study time period (Figure 1). However, the seventh year of the study showed the first peak (with 26 victims or 10.8%) over the second half of the period. Most fatal crashes occurred among bus/minibus users in the fourth year of study (21 March 2009-20 March 2017) with 43 victims (17.8%).

### Demographics

**Figure 2:**
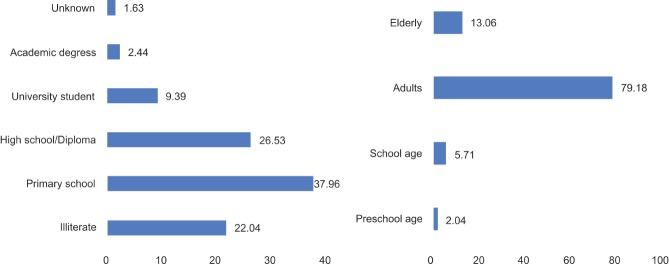
Distribution of Bus and Minibus Users’ Mortalities by Schooling Level (left) and Age Distribution (right) in East Azerbaijan (March 2006-March 2017)

The BuMUs mortalities among men were 70%, twice more than women mortalities. The mean age was 41.5 years (SD: 18.6). According to the Figure 2 (right), of 22% illiterate victims, 2.2% were under six years old, and the remaining were seven and above. More than a third of BuMUs had diploma/high school education. As shown in Figure 2 (left), the majority of victims were working human force, specifically adults at 18 to 64 years of age, followed by elderly (≥ 65 years old) with a mortality of 79.2% and 13% respectively. The rest of the victims were at their school (7 to 17) and preschool age (≤6) (2%).

The majority of victims were self-employed (25.3%), followed by housekeepers (17%) and drivers (12.9%). The highest percentage of adult fatalities were self-employed individuals (27.4%) then housekeepers and drivers with 16.8% and 15.3%, respectively. The job for under six-year-olds was defined as “others” with 2% fatalities.

### Crash Mechanisms

A comparative study of crash mechanisms between bus and minibus users (table 1) shows that the most common crash mechanisms were a collision with another vehicle that was followed by a rollover. The frequency of rollover by bus was significantly higher than minibus (P-value<0.01), whereas crashes causing the fall of the vehicle was increased for minibuses. Over the studied twelve years, around five percent of bus/minibus user fatalities occurred due to crashing into fixed objects. The minibus-pedestrian crash was more prevalent than bus-pedestrian crash. Lorry, van or trailer was the leading crash counterpart that comprised more than 30% of cases (Table 1). Excluding the cases when no other vehicle was engaged, lorries, vans or trailers caused 59% of deaths that was followed by cars causing 16% of the fatalities and pickups with 10%. Minibuses were crashed into by cars, pickups and other minibuses more than buses (P-value <0.05).

As seen in Table 1, the leading cause of death was head trauma accounting for more than half of cases, followed by multiple fractures (about 14%), mixed causes (11.3%), and then bleeding (8.6%). There was a statistically significant difference between the type of main cause of death and the type of vehicle used (bus/minibus).

As seen in Table 2, the leading cause of death was head trauma accounting for more than half of cases, followed by multiple fractures (about 14%), mixed causes (11.3%), and then bleeding (8.6%). There was a statistically significant difference between the type of main cause of death and the type of vehicle used (bus/minibus).

### Variables Associated with Hospital Mortality

Logistic regression analysis was done to determine the variables associated with occurring death at the hospital among bus and minibus users in models. The results are illustrated in Table 3. In the analysis, pre-hospitalization death was categorized as null and post-hospitalization death was one. Two variables were statistically significant in the model. Victims were 4.17 times more likely to die at the hospital when the crash occurred on inner city roads (95% CI: 1.7-9.9). The elderly were 2.78 times more likely to die at the hospital when compared to other age groups (95% CI: 1.23-6.26)

### Other Important Results

Regarding the role of victims at the accident time, 188 (76.7%) of the victims were passengers and 53 (21.6%) were drivers.

**Figure 3:**
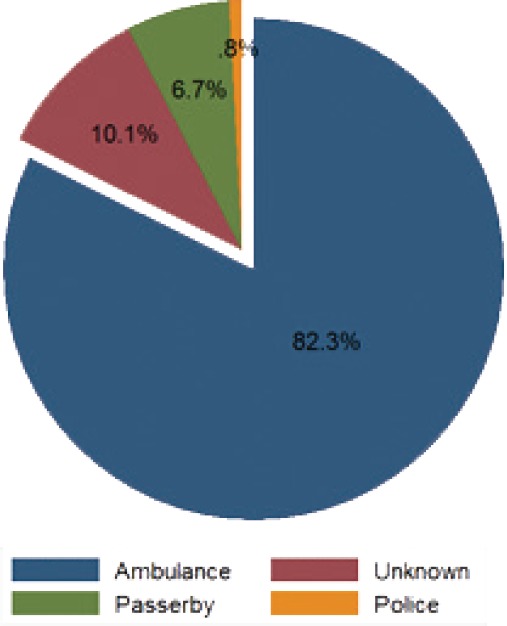
Transport mode of injured bus/minibus users to hospitals in East Azerbaijan (March 2006-March 2017)

Regarding Figure 3, data analysis showed that 74% of users died at the scene or at the pre-hospital stage. Emergency Medical Services (EMS) act as the main mode of transferring bus/minibus victims to hospitals. Only 6.7% of victims were transferred by police. The role of pedestrians and police in transporting victims has declined to null since 2014, but they had previously played a role in transporting RTIs victims, especially passersby.

**Table 1:** Distribution of variables compared between vehicles used when crashes in East Azerbaijan (March 2006-March 2017)

Variables	Type of vehicle used		Total	P-value*
	Bus	Minibus		
**Crash mechanism**				
Vehicle-Vehicle	70 (49.6%)	69 (69%)	139 (57.7%)	<0.01
Rollover	51 (36.2%)	14 (14%)	65 (26.9%)	<0.01
Fall	8 (5.7%)	7 (7%)	15 (6.2%)	0.67
Vehicle-Object	6 (4.3%)	6 (5%)	12 (4.9%)	0.54
Others	5 (3.5%)	1 (1%)	6 (2.5%)	0.21
Vehicle-Pedestrian	1 (0.7%)	3(3%)	4 (1.7%)	0.21
Total	141 (100%)	100 (100%)	241(100%)	
**Crash counterpart vehicle**				
Lorry, van or trailer	48 (33.3%)	36 (38.3%)	84 (35.3%)	<0.05
Car	10 (6.9%)	13 (13.8%)	23 (9.7%)	
Pickup	7 (4.8%)	8 (8.5%)	15 (6.3%)	
Bus	4 (2.8%)	1 (1.1%)	5 (2.1%)	
Minibus	1 (0.7%)	5 (5.3%)	6 (2.5%)	
Other vehicles	2 (1.2%)	1 (1.1%)	3 (1.3%)	
Unknown	3 (2.1%)	4 (4.3%)	7 (2.9%)	
No crash counterpart	69 (47.9%)	26 (27.7%)	95 (39.9%)	
Total	144 (100%)	94 (100%)	238 (100%)
**Main cause of death**				
Head Trauma	81 (56.2%)	68 (68%)	149 (61%)	0.04
Mixed causes	21 (14.6%)	24 (24%)	45 (18.4%)	0.04
Multiple Fractures	21 (14.6%)	5 (5%)	26 (10.6%)	0.01
Bleeding	12 (8.3%)	2 (2%)	14 (5.7%)	0.03
Others	9 (6.2%)	1 (1%)	10 (4%)	0.65
Total	144 (100%)	100 (100%)	244 (100%)	

**Table 2:** Distribution of main cause of death compared between vehicles crashed in East Azerbaijan (March 2006-March 2017)

Main cause of death	Type of vehicle used		Total	P-value*
	Bus	Minibus		
Head Trauma	81 (56.2%)	68 (68%)	149 (61%)	0.04
Mixed causes	21 (14.6%)	24 (24%)	45 (18.4%)	0.04
Multiple Fractures	21 (14.6%)	5 (5%)	26 (10.6%)	0.01
Bleeding	12 (8.3%)	2 (2%)	14 (5.7%)	0.03
Others	9 (6.2%)	1 (1%)	10 (4%)	0.65
Total	144 (100%)	100 (100%)	244 (100%)	

* P-values are according to chi-squared test and to examine if proportions of main cause of death are different between the bus and minibus users’ fatalities.

**Figure 4:**
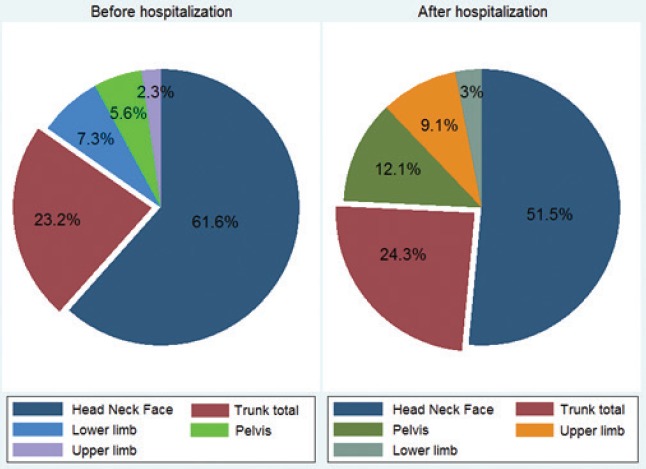
Distribution of injured body organs compared between bus/minibus users who died before/after hospitalization in East Azerbaijan (March 2006-March 2017)

Head-neck-face trauma was the main type of injury sustained in 61.6% of the victims who died prior to hospitalization versus 51.5% of those who died post-hospitalization (Figure 4). However, injuries to upper limbs and pelvis were more prevalent among those bus/minibus users who died after admission to the hospital. The same percentage of users suffered injuries to the chest, abdomen and column vertebrae (those categorized as total trunk) at the scene or pre- or post-hospitalization.

**Table 3:** Multivariate Binary logistic analysis for determinants of hospital deaths among bus/minibus users, East Azerbaijan (March 2006-March 2017)

Determinants	OR*	P-value	95% CI**	
**Elderly**	2.78	0.013	1.23705	6.263501
**Inner city roads**	4.17	0.001	1.750852	9.948354

* Odds Ratio

** Confidence Interval

## Discussion

The results showed that out of 11016 deaths of RTIs registered in EAFMD between 2006 and 2017, 245 mortalities belonged to bus and minibus users (BuMUs). Despite the low costs of using the public transportation system (especially that of buses and minibuses) in Iran, people are still less interested in utilizing this system of transportation due to their lower comfort and speed than that of personal cars [8]. So, we can conclude that, despite the lower utilization of the bus and minibus in Iran, the mortality rate among users is significant. ERSO (European Road Safety Observatory) reported that 32% of the deaths of pedestrians and cyclists in the European Union were related to buses and similar vehicles [9]. Also, comparing RTIs per 100,000 Iranian inhabitants (747) to that of other countries like the Azerbaijan Republic (28), Turkmenistan (28), Turkey (94), Australia (534), the United States (675), and the UK (370) revealed the necessity of paying more attention to RTAs in Iran [10]. Highly populated and dense provinces had less share of roads. Throughout ten years after 1997 and up to 2006, the average life of the public transportation means was 16 years for buses, 20 years for minibuses and 20.4 years for heavy trucks, on inter-city or inter-village ways. Moreover, regarding rail transport, the amount of goods transported by this system is greatly lower when compared to that of roads [11]. Therefore, it can be concluded that considering the high depreciation of Iranian trucks and buses, the increased number of RTAs among this type of transporting vehicles is not an unexpected issue.

Considering the results, almost 88% of mortalities among BuMUs happened on outer-city roads and crashes on outer-city roads were discovered as a predictive factor for pre-hospital mortality and inner-city crashes for post-hospitalization deaths because users were 4.17 times more likely to die at the hospital when the crash occurred on inner city roads. The reasonable explanation could be providing on-time EMS services. Several studies revealed a shorter time of EMS response to inner-city crashes than to interurban ones [12, 13]. However, their death in the hospital or later at home may be due to side effects of injuries or lack of appropriate care. Also, it could be assumed that inner-city injuries are less deadly because of vehicles’ low speed. High speeds by intercity buses cause more injuries in car crashes [14]. In the United States state of Iowa, catastrophic school bus crashes happen with high speed, usually above 88 km/h [15]. In the present study, the elderly were 2.78 times more likely to die at the hospital when compared other age groups in East Azerbaijan. A similar outline is stated in a study on fatal RTIs among the elderly [16] and car users [17]. Despite being cared for earlier and more than other age groups of bus/minibus users and their faster transportation to the hospital, the elderly decease more at the hospital level. Several causes are involved in this regard. The probability of an elderly accident on the inner-city roads is higher than outer-city. Also, in the elderly group, the time to reach the hospital in inner-city crashes is lower than the outer-city because the distance from the hospital to the accident scene is short. Additionally, the underlying problems and other associated illnesses that the elderly suffer from are the cause of their high mortality in hospitals [16].

The study revealed that crashing with another vehicle and rolling over were the most common crash mechanisms for buses and minibuses during the last twelve years. However, vehicle-vehicle crashes were significantly higher when talking about minibuses. Due to their large size and weight, buses give passengers more protection than other road transport vehicles such as minibuses and other cars. In the study site, inner-city buses were less exposed to crashes with other vehicles. The existence of a unique bus line is likely to lead to a reduction in injuries given that it can act as a roadside buffer and thus reduce vehicle-vehicle crashes and vehicle with other roadside factors [18]. Results showed that head trauma caused about three-fifths of BuMUs mortalities [19], either among bus or minibus users. However, the main causes of death among bus users differ from those among minibus users. Head trauma and mixed causes were significantly higher among minibus users, while multiple fractures and bleeding were higher among bus users. This result could stem from the usually higher speed rate of vehicles. Buses are usually driven at a lower speed, especially inner-city buses. In LMICs, it is important to provide safe buses since the bus transportation system plays a fundamental role in providing affordable and adequate means of transportation for the majority of the population and it contributes to urban and rural development [20].

In this study, the main injuries sustained by victims were head-neck-face traumas, pre- and post-hospitalization. These were followed by total trunk injury estimated to be found in a fourth of the total number of cases. Lack of a seat belt for passengers may cause an increase in the rate of injuries to the head, neck and face. Almost 74% of BuMUs drivers died during the pre-hospital stage. This finding is in line with other studies suggesting the great number of RTAs mortalities in LMICs [21] and Iran [22, 23] that occurred before reaching the hospital. By providing adequate emergency and paramedic services, this number can be extensively reduced [24]. Another study suggested providing comprehensive trauma systems [25] that should be greatly considered in the Iranian setting [26]. According to demographics, considering the higher rate of BuMUs mortalities among men than women that is reported to be 70%, other numerous studies suggested that men suffer three times more from road injuries than women in Iran [27–29]. In similar countries to Iran where women play a smaller role in transportation and driving and they are mostly passengers/occupants, so such gender disparities can ordinarily be probable [17]. Also, educational stage was an influential factor in RTAs’ occurrence as the greatest bulk of mortalities happened among less literate (37%) or illiterate (22%) BuMUs. It is in line with an Iranian study [30] that showed a higher chance of being involved in RTAs among illiterate or less literate drivers with less prestigious occupations. However, education had little impact on accident involvement among Dutch and Taiwanese drivers [29], but for Chinese drivers, the issue was reversed [31]. According to the victims’ job situation, the majority of users were self-employed. Most of them were passengers, showing that people with less prestigious occupations often use bus/minibus to commute or travel for their occupational affairs and they are generally tired at the time of the crashes, bearing the consequences of such unsafe conditions of transport. Additionally, there are socioeconomic factors affecting the issue and are more highlighted in LMICs. The level of income is associated with the job position. People with a low level of income often walk or travel by bus or by cycling, so they are more prone to RTAs [32]. Among Sri Lankan drivers, the rate of bus injuries is closely related to working conditions, salary incentives and job satisfaction [33]. There is no doubt that driver-related characteristics have a significant relationship with the rate of bus collisions [34]. In line with the studies on the health of bus drivers, this study suggests that by concentrating on improving worker health (including improving physical and mental health of drivers), reducing fatigue and reducing alcohol intake, tobacco and drug use, a reduction in the accident rate in buses, Job-shift staffing and driver absences can be predicted [35]. The quality of patient care is affected by many factors, one of the most important issues in this regard being the increase in drug costs [36–38]. The merit of the present study is that it is one of the first Iranian studies aiming at examining a variety of epidemiological characteristics and possible reasons for BuMUs mortalities over 12 years in the East Azerbaijan province of Iran.

## Conclusion

It appears that low literacy, the presence of younger drivers as well as non-standard roads and improper safety of road infrastructure for bus/minibus vehicles are the problems that must be taken into consideration at the time of planning and policy making. The type of vehicle in terms of safety conditions can also be considered in the mortality rates associated with crashes by bus / minibus, which requires further research in this field.

## Acknowledgment

This work is part of the “Integrated Road Traffic Injury Registry (IRTIR)” project [39]. The authors acknowledge any support given by the Forensic Medicine Organization of the province East Azerbaijan.

## Conflict of Interest

The authors confirm that there are no conflicts of interest.
